# Supplementary material for: Understanding the Gastrointestinal Behavior of the Coffee Pulp Phenolic Compounds under Simulated Conditions
Source: Antioxidants (Basel). 2022 Sep 15;11(9):1818. doi: 10.3390/antiox11091818 (PMC9495553; doi:10.3390/antiox11091818)
Supplement: Supplementary file 1 [file antioxidants-11-01818-s001.zip › antioxidants-1897861-supplementary.pdf]

Understanding the gastrointestinal behavior of the coffee pulp phenolic compounds under simulated conditions

Silvia Cañas[1,2], Miguel Rebollo-Hernanz[1,2], Cheyenne Braojos[1,2], Vanesa Benítez[1,2], Rebeca Ferreras-Charro[3], Montserrat Dueñas[3], Yolanda Aguilera[1,2], María A. Martín-Cabrejas[1,2]

[1] Department of Agricultural Chemistry and Food Science, Faculty of Science, C/ Francisco Tomás y Valiente, 7. Universidad Autónoma de Madrid, 28049, Madrid, Spain.

[2] Institute of Food Science Research (CIAL, UAM-CSIC). C/ Nicolás Cabrera, 9. Universidad Autónoma de Madrid, 28049, Madrid, Spain.

[3] Grupo de Investigación en Polifenoles, Unidad de Nutrición y Bromatología, Facultad de Farmacia, Universidad de Salamanca, Campus Miguel de Unamuno, 37007 Salamanca, Spain

Table S1. Retention index, bioaccessibility, and potential bioavailability (%) of phenolic families from coffee pulp flour (CPF) and extract (CPE) after *in vitro* digestion.

Compounds	Retention Index		Bioaccessibility		Bioavailability	
	OP	GP	IP	CP	C2A	HIA
**Coffee Pulp Flour**						
Phenolic acids						
Total Hydroxybenzoic acids	$61.1 \pm 2.7^{abc***}$	$70.3 \pm 2.5^{ab**}$	79.0 ± 12.6^{a}	$53.9 \pm 6.5^{abc*}$	66.9 ± 10.7^{abc}	48.6 ± 7.8^{c}
Total Hydroxycinnamic acids	$50.7 \pm 2.5^{b***}$	71.1 ± 7.2^{a}	$82.3 \pm 11.4^{a**}$	$46.6 \pm 7.1^{b***}$	$12.4 \pm 1.7^{c**}$	$22.8 \pm 3.1^{c**}$
**Total Phenolic acids**	$\mathbf{57.7 \pm 2.7^{bc***}}$	$\mathbf{70.6 \pm 5.8^{ab*}}$	$\mathbf{80.1 \pm 12.2^{a*}}$	$\mathbf{51.5 \pm 6.7^{cd**}}$	$\mathbf{48.8 \pm 7.9^{cd}}$	$\mathbf{40.0 \pm 6.3^{d}}$
Flavonoids						
Total Flavones	$27.8 \pm 1.5^{c**}$	$53.3 \pm 7.1^{a*}$	41.9 ± 6.8^{b}	31.3 ± 3.5^{c}	5.0 ± 0.8^{d}	7.8 ± 3.1^{d}
Total Flavonols	$38.9 \pm 3.3^{b***}$	$61.8 \pm 8.3^{a**}$	58.7 ± 8.9^{a}	12.5 ± 1.9^{c}	14.1 ± 2.4^{c}	13.6 ± 2.2^{c}
**Total Flavonoids**	$\mathbf{35.8 \pm 2.8^{b***}}$	$\mathbf{59.4 \pm 7.9^{a**}}$	$\mathbf{54.0 \pm 8.3^{a}}$	$\mathbf{17.7 \pm 2.3^{c}}$	$\mathbf{11.6 \pm 1.9^{c}}$	$\mathbf{12.0 \pm 1.9^{c}}$
**Total Phenolics**	$\mathbf{56.2 \pm 2.7^{bc***}}$	$\mathbf{69.8 \pm 5.9^{ab*}}$	$\mathbf{78.4 \pm 12.0^{a}}$	$\mathbf{49.3 \pm 6.5^{cd**}}$	$\mathbf{46.4 \pm 7.6^{cd}}$	$\mathbf{38.2 \pm 6.1^{d*}}$
**Coffee Pulp Extract**						
Phenolic acids						
Total Hydroxybenzoic acids	$105.5 \pm 8.2^{a***}$	91.8 ± 10.9^{ab}	63.8 ± 9.3^{cd}	78.7 ± 12.0^{bc}	53.8 ± 7.9^{de}	39.1 ± 5.7^{e}
Total Hydroxycinnamic acids	104.5 ± 5.3^{a}	84.3 ± 8.2^{b}	52.3 ± 2.5^{c}	104.4 ± 12.7^{a}	7.7 ± 0.3^{d}	14.5 ± 0.8^{d}
**Total Phenolic acids**	$\mathbf{105.2 \pm 7.2^{a}}$	$\mathbf{89.3 \pm 10.0^{a}}$	$\mathbf{60.0 \pm 7.0^{b}}$	$\mathbf{87.1 \pm 12.3^{a}}$	$\mathbf{38.7 \pm 5.2^{c}}$	$\mathbf{31.1 \pm 4.0^{c}}$
Flavonoids						
Total Flavones	87.5 ± 25.8^{a}	86.5 ± 22.5^{a}	–	–	–	–
Total Flavonols	105.4 ± 8.2^{a}	95.3 ± 10.6^{a}	–	–	–	–
**Total Flavonoids**	$\mathbf{100.9 \pm 13.1^{a}}$	$\mathbf{93.0 \pm 13.8^{a}}$	**–**	**–**	**–**	**–**
**Total Phenolics**	$\mathbf{105.0 \pm 7.5^{a}}$	$\mathbf{89.9 \pm 10.2^{ab}}$	$\mathbf{56.9 \pm 6.7^{c*}}$	$\mathbf{82.6 \pm 11.8^{b}}$	$\mathbf{36.7 \pm 5.0^{d}}$	$\mathbf{29.4 \pm 3.8^{d}}$

Results are reported as mean ± SD ($n = 3$). Mean values within a line followed by different superscript letters (a, b, c, d, e) are significantly different when subjected to Tukey's test ($p < 0.05$). OP: Oral Phase; GP: Gastric Phase; IP: Intestinal Phase; CP: Colonic Phase; C2A: Caco-2 Absorption; HIA: Human Intestinal Absorption. Mean values followed by superscript asterisks significantly differ (CPF *vs*. CPE) when subjected to *T*-test (* $p < 0.05$, ** $p < 0.01$, *** $p < 0.001$).

Table S2. Physicochemical properties and intestinal absorption of the phenolic metabolites from the coffee pulp *in silico* colonic metabolism.

Phenolic Compound	Common name	Abbreviation	M_W	LogP	C2A	HIA
Quercetin 3,7-diglucoside	–	Q3,7G	626.5	−3.07	11.0 ± 2.5	7.6 ± 3.1
Quercetin 3-rutinoside	Rutin	RUT	610.5	−1.69	13.7 ± 3.5	22.2 ± 1.2
Apigenin 6,8-*C*-diglucoside	Vicenin-2	VIC2	594.5	−2.39	11.9 ± 3.9	18.6 ± 4.0
3,5-Dicaffeoylquinic acid	Isochlorogenic acid A	3,5-dCQA	516.4	1.03	10.2 ± 3.7	34.2 ± 9.7
Quercetin 3-glucoside	Isoquercetin	Q3G	464.4	−0.54	44.1 ± 12.3	31.7 ± 16.2
Quercetin 7-glucoside	Quercimeritrin	Q7G	464.4	−0.54	44.5 ± 13.1	24.6 ± 9.1
Apigenin 6-*C*-glucoside	Isovitexin	API6G	432.4	0.09	24.2 ± 5.0	45.5 ± 19.2
3-*O*-Caffeoylquinic acid	Chlorogenic acid	3-CQA	354.3	−0.65	15.3 ± 3.4	34.4 ± 1.9
4-*O*-Caffeoylquinic acid	Cryptochlorogenic acid	4-CQA	354.3	−0.65	15.2 ± 3.8	26.3 ± 6.2
5-*O*-Caffeoylquinic acid	Neochlorogenic acid	5-CQA	354.3	−0.65	15.3 ± 3.4	34.4 ± 1.9
5-*p*-Coumaroylquinic acid		5-CouQA	338.3	−0.35	24.7 ± 5.8	38.1 ± 5.9
3-(3,4-Dihydroxyphenyl)-1-(2,4,6-trihydroxyphenyl)propane-1,2-dione	Dihydroquercetin chalcone	dhQCH	304.2	1.21	30.3 ± 4.0	44.3 ± 13.9
3,3',4',5,7-Pentahydroxyflavanone	Dihydroquercetin	dhQ	304.2	1.19	60.3 ± 20.2	54.3 ± 10.5
3,3',4',5,7-Pentahydroxyflavone	Quercetin	Q	302.2	1.99	36.6 ± 3.9	60.5 ± 16.7
3-(3-Hydroxyphenyl)-1-(2,4,6-trihydroxyphenyl)propane-1,2-dione	–	THdhFLCH	288.2	1.50	53.7 ± 8.7	45.5 ± 13.1
3,3',5,7-Tetrahydroxyflavanone	–	THdhFL	288.2	1.48	80.6 ± 8.9	53.0 ± 6.0
3-(4-Hydroxyphenyl)-1-(2,4,6-trihydroxyphenyl)propane-1,2-dione	Dihydrokaempferol chalcone	dhKMPCH	288.2	1.50	49.2 ± 12.4	47.7 ± 14.6
3,4',5,7, -Tetrahydroxyflavanone	Dihydrokaempferol	dhKMP	288.2	1.48	81.0 ± 8.7	53.0 ± 6.0
3,4',5,7-Tetrahydroxyflavone	Kaempferol	KMP	286.2	2.28	72.8 ± 13.0	60.6 ± 13.6
3,3',5,7-Tetrahydroxyflavone	–	THFL	286.2	2.28	72.9 ± 12.6	63.9 ± 16.9
2',4,4',6'-Tetrahydroxydihydrochalcone	Phloretin	PT	274.3	2.32	61.1 ± 18.3	51.6 ± 8.9
3-Phenyl-1-(2,4,6-trihydroxyphenyl)-1,2-propanedione	Dydrogalangin chalcone	dhGLCH	272.2	1.80	65.7 ± 17.6	49.3 ± 16.2
4',5,7-trihydroxyflavanone	Naringenin	NAR	272.2	2.51	96.9 ± 1.8	71.7 ± 19.7
2',4,4',6'-Tetrahydroxychalcone	Naringenin chalcone	NARCH	272.2	2.41	62.7 ± 17.6	54.1 ± 6.9
3,5,7-Trihydroxyflavanone	Dihydrogalangin	dhGL	272.2	1.78	96.1 ± 2.1	69.1 ± 22.1
3,5,7-Trihydroxyflavone	Galangin	GL	270.2	2.58	95.1 ± 2.4	70.5 ± 23.5
4',5,7-Trihydroxyflavone	Apigenin	API	270.2	2.58	95.9 ± 2.2	73.2 ± 20.1
2',4',6'-Trihydroxydihydrochalcone	Dihydrochrysin chalcone	dhCHRYCH	258.3	2.62	95.9 ± 2.2	67.0 ± 24.3

5,7-Dihydroxyflavanone	Pinocembrin	PB	256.2	2.80	96.3 ± 2.0	71.6 ± 19.6
2',4',6'-Tetrahydroxychalcone	Pinocembrin chalcone	PBCH	256.2	2.70	96.0 ± 2.2	69.2 ± 22.0
5,7-Dihydroxyflavanone	Dihydrochrysin	dhCHRY	256.2	2.80	96.9 ± 1.7	72.2 ± 20.2
5,7-Dihydroxyflavone	Chrysin	CHRY	254.2	2.87	95.6 ± 2.3	73.4 ± 20.3
3,4-Dihydroxyphenylpyruvic acid	–	3,4-HPPyA	196.2	0.29	66.9 ± 12.2	43.8 ± 3.1
3,4-Dihydroxydihydrocinnamic acid	Dihydrocaffeic acid	dhCA	182.2	1.12	91.8 ± 3.0	57.4 ± 12.0
3,4-Dihydroxycinnamic acid	Caffeic acid	CA	180.2	1.20	80.5 ± 8.3	64.5 ± 8.9
3-Hydroxyphenylpyruvic acid	–	3-HPPyA	180.2	0.59	92.3 ± 3.0	60.2 ± 17.2
4-Hydroxyphenylpyruvic acid	–	4-HPPyA	180.2	0.59	93.4 ± 2.8	60.6 ± 16.4
3,4,5-Trihydroxybenzoic acid	Gallic acid	GA	170.1	0.50	53.9 ± 6.6	43.2 ± 0.1
2,4,6-Trihydroxybenzoic acid	Phloroglucinic acid	PGA	170.1	0.50	51.6 ± 8.9	38.2 ± 8.4
4-Hydroxy-3-methoxybenzoic acid	Vanillic acid	VA	168.1	1.10	93.2 ± 2.9	71.3 ± 7.6
3,4-Dihydroxyphenylacetic acid	Homoprotocatechuic acid	3,4-HPAA	168.1	0.72	90.7 ± 3.1	57.0 ± 7.7
3-(3-Hydroxyphenyl)propionic acid	*m*-Hydrocoumaric acid	3,3-HPPA	166.2	1.41	96.9 ± 1.8	79.2 ± 12.2
3-(4-Hydroxyphenyl)propionic acid	Phloretic acid	3,4-HPPA	166.2	1.41	97.1 ± 1.7	81.1 ± 12.6
4-Hydroxycinnamic acid	*p*-Coumaric acid	*p*-CouA	164.2	1.49	97.0 ± 1.7	82.8 ± 8.3
3-Hydroxycinnamic acid	*m*-Coumaric acid	*m*-CouA	164.2	1.49	96.9 ± 1.8	82.8 ± 8.3
Phenylpyruvic acid	–	PPyA	164.2	0.88	97.5 ± 1.5	74.7 ± 6.3
3,4-Dihydroxybenzoic acid	Protocatechuic acid	PCA	154.1	0.80	87.4 ± 4.5	63.1 ± 8.0
3,5-Dihydroxybenzoic acid	α-Resorcylic acid	α-RA	154.1	0.80	89.9 ± 3.5	61.4 ± 14.0
3-Hydroxyphenylacetic acid	–	3-HPAA	152.5	1.02	96.9 ± 1.8	76.0 ± 6.1
3-Methoxybenzoic acid	*m*-Anisic acid	*m*-AnA	152.1	1.39	97.7 ± 1.3	91.4 ± 8.7
4-Hydroxyphenylacetic acid	–	4-HPAA	152.1	1.02	96.8 ± 1.8	79.1 ± 7.2
3-Phenylpropionic acid	Hydrocinnamic acid	hCiA	150.2	1.70	99.0 ± 0.6	90.5 ± 4.5
Cinnamic acid	–	CiA	148.2	1.78	98.8 ± 0.7	91.8 ± 2.3
4-Hydroxybenzoic acid	*p*-Salicylic acid	*p*-SA	138.1	1.09	97.1 ± 1.7	79.3 ± 4.7
3-Hydroxybenzoic acid	*m*-Salicylic acid	*m*-SA	138.1	1.09	96.9 ± 1.8	76.8 ± 2.2
Phenylacetic acid	–	PAA	136.1	1.31	99.0 ± 0.6	86.5 ± 1.1
Benzoic acid	–	BA	122.1	1.38	99.2 ± 0.5	94.6 ± 4.7

M_w: molecular weight; logP: partition coefficient; C2A: Caco-2 Absorption; HIA: Human Intestinal Absorption

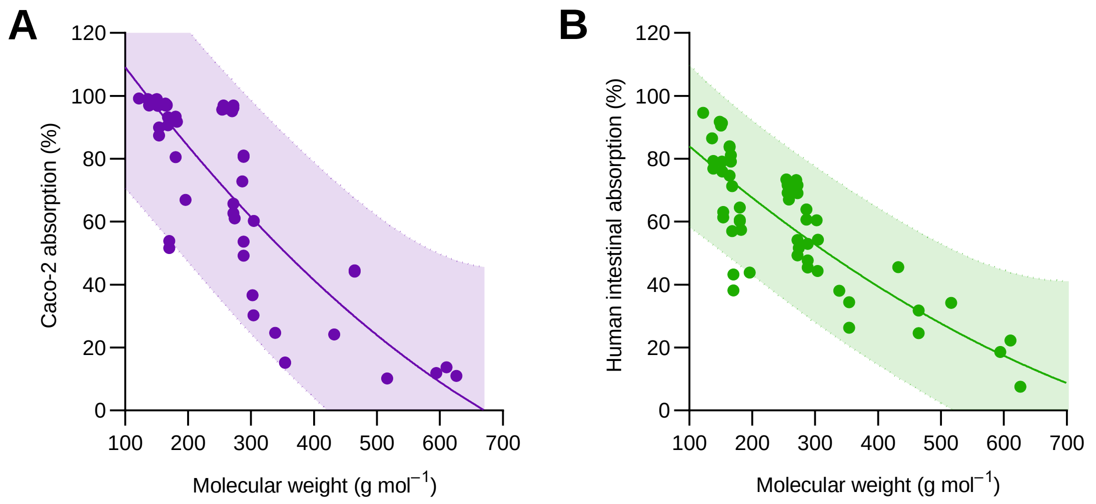

Figure S1. Association between the molecular weight of the coffee pulp phenolic metabolites and their Caco-2 (**A**) and human intestinal (**B**) absorption.
